# Biomechanical evaluation of percutaneous cement discoplasty by finite element analysis

**DOI:** 10.1186/s12891-022-05508-1

**Published:** 2022-06-20

**Authors:** Hongwei Jia, Bin Xu, Xiangbei Qi

**Affiliations:** Department of Orthopaedic Surgery, The Third Hospital of Hebei Medical University, Hebei province, Shijiazhuang, China

**Keywords:** Percutaneous cement discoplasty, Indirect decompression effect, Biomechanical evaluation, Finite element analysis

## Abstract

**Background:**

Percutaneous cement discoplasty (PCD) is a minimally invasive treatment for degenerative lumbar spine disease, but the relationship between decompression effect on the nerve root and different doses of bone cement is uncertain.

**Purpose:**

To investigate the indirect decompression effect of cement with different doses on nerve roots and the biomechanical changes on the spine during PCD using finite element analysis (FEA).

**Methods:**

FEA was adapted to analyze the mechanical changes in the lumbar vertebrae before and after the application of PCD.CT scan images of adult males were utilized to establish a finite element model of the lumbar vertebral body using mimics and Pro/E software. The images were divided into four models: the normal model (normal, model N), the disc degeneration model (high, model H), the intervertebral disc injected with 3 mL of bone cement (model H1), and the intervertebral disc injected with 5 mL of bone cement (model H2). All models were analyzed using the ABAQUS6.14.2 software. The normal physiological movements were simulated, and the mechanical changes in the lumbar vertebrae were observed prior to and after the cement filling application.

**Results:**

The stress of the nerve root in model H was the largest. The nerve root stress in the model H2 was the smallest during flexion, extension, left bending, right bending, left rotation, and right rotation at 90%, 44%, 25%, 56%, 56%, and 51% of the normal benchmark, respectively. After the injection of bone cement, the nerve root stress is reduced. The greater the amount of cement, the lesser the nerve root stress. The motion was reduced in models H, H1, and H2, and there were differences between models H1 and H2. Cartilage endplate stress was less in model H2 than in model H1.

**Conclusions:**

The nerve root stress increased after degeneration and decreased after intervertebral height recovery through cement injection, resulting in a significant indirect decompression effect.The stress of the nerve root decreased with the increase in the amount of cement injection.

## Introduction

Degenerative disc diseases are gradually emerging as the major aging-related condition affecting the health of the elderly throughout the world. Degenerative disorders of the spine, such as lumbar disc herniation that cause lower back pain, are becoming increasingly common in the elderly. Treatment approaches for clinically symptomatic patients affected by these conditions often include traditional approaches for managing the symptoms. In contrast, patients who fail to respond to traditional disease management often require surgical treatment. One of the major challenges encountered by orthopedic clinicians is locating the source of the lumbar and lower extremity pain and selecting the appropriate treatment modality.

Moreover, aging-related clinical conditions, such as severe osteoporosis, severe spinal deformities, and cardio-cerebrovascular disease, further limit the surgical modalities to be selected. In cases of severe spinal deformity or degenerative spinal instability, traditional open surgery often leads to a high rate of complications, such as wound infection [1, 2]. Medical comorbidities associated with degenerative spine disorders, such as cardiopulmonary disease, advanced rheumatic disease, diabetes, or osteoporosis, increase the perioperative risk. Traditional procedures are traumatic, result in greater bleeding, and have a longer operative duration. In comparison, minimally invasive surgery has the advantages of a lower operative duration, less intraoperative blood loss, lower infection rate, reduced duration of hospital stay, and reduced costs [3, 4]. Minimally invasive techniques are, therefore, being considered as a suitable treatment option by clinicians and patients.

In orthopedics, bone cement technology is used widely for joint replacement, vertebroplasty, and other treatment approaches [5, 6]. With further progress in this technology, the scope of application of bone cement material has extended to treating degenerative spine diseases and spinal deformity correction [7–10]. One of the minimally invasive techniques that utilize bone cement is percutaneous cement discoplasty (PCD), which was developed originally by Varga for the treatment of degenerative disc pathologies using polymethylmethacrylate (PMMA) as a filler into the intervertebral disc to achieve intervertebral space elevation and the indirect decompression of the nerve root. In 2015, Varga et al. used bone cement in a percutaneous cement discoplasty (PCD) surgery. They reported that the elderly of all patients with degenerative disc disease experienced significantly less postoperative lower back pain. At the 6^th^-month follow-up, over 50% of the patients were reported to have at least a 10-point decrease in the Oswestry disability index score [11]. Since then, the PCD technique has gradually been recognized by many clinicians as an effective approach to treat disc degenerative disease. Several scholars have used the PCD technique to treat patients with degenerative scoliosis and spinal diseases, such as symptomatic lumbar disc herniation treated by lumbar discectomy combined with PCD, which can achieve satisfactory clinical outcomes [12, 13]. However, the biomechanical studies analyzing how the bone cement acts on the disc after application are fewer compared to clinical studies. Techens et al. performed flexion extension, extension, and lateral bending tests for lumbar disc shaping in pigs. They reported that discoplasty restored the post-disc height and could open neural foramina, although there was no effect on the mobility or rigidity of the spine [14]. On the contrary, in another study, cement injection into the intervertebral disc of cadaveric specimens increased the occurrence of axial spinal rigidity [15]. The biomechanical changes in spinal mobility after the bone cement acts on the disc, the effects on adjacent endplates, nerve root stresses, and whether the cement application increases the risk of adjacent vertebral fractures remain to be elucidated.

Finite element analysis is applied widely in biomechanical research on the human spine. This method enables more realistic mimicking of the various physiological states than animal models and cadaveric specimen studies [16–18]. According to our knowledge, few studies have applied finite element analysis for studying the biomechanical alterations of the spine after PCD. Therefore, the present study aimed to apply the method of finite element analysis to explore the effects of PCD on the mechanical properties of the spine.

## Materials and methods

### L1-S1 3D geometry model building

A 28-year-old healthy young male volunteer(weight 65 kg, height 173 cm)with no previous history of lumbar trauma and disease was selected for the study. The X-ray imaging analysis excluded any other spinal disorder, after which consecutive whole spine scans were performed (matrix 512 × 512; layer distance and layer thickness was 0.625 mm). A 1-mm-thick serial tomography scan along the transverse aspect of the lumbar 1 vertebral body was performed together with a transaxial spiral CT scan. The resulting tomographic images were exported to DICOM (digital imaging and communications in medicine) format to the computer.

### Establishment of the digital model

The 3D models, such as vertebral bodies and ligaments, were established. The data obtained from the CT scan were transferred to a computer in DICOM format using the mimics 11.1 software. The geometric model of the lumbar vertebral body and ligaments was established and subsequently entered into the Pro/E software for processing the model geometry. Since there is a low content of cancellous bone within the pedicles and spinous processes, it was considered that cancellous bone mainly existed in the vertebral bodies and, accordingly, a 1-mm-thick cortex was simulated in the vertebral model. The ligaments were scaffolding based on the scanning data. ANSA14.2 software was employed to construct a finite element mesh model of the normal lumbar spine structure, in combination with ABAQUS6.14.2 to define the connections, boundary conditions, applied loads, etc. The pre-treatment contents were completed in ANSA14.2. ABAQUS/standard implicit solver was employed for solving. ABAQUS6.14.2 post-processing viewer was used to analyze the results.

### Establishment of the experimental model

**Fig. 1:**
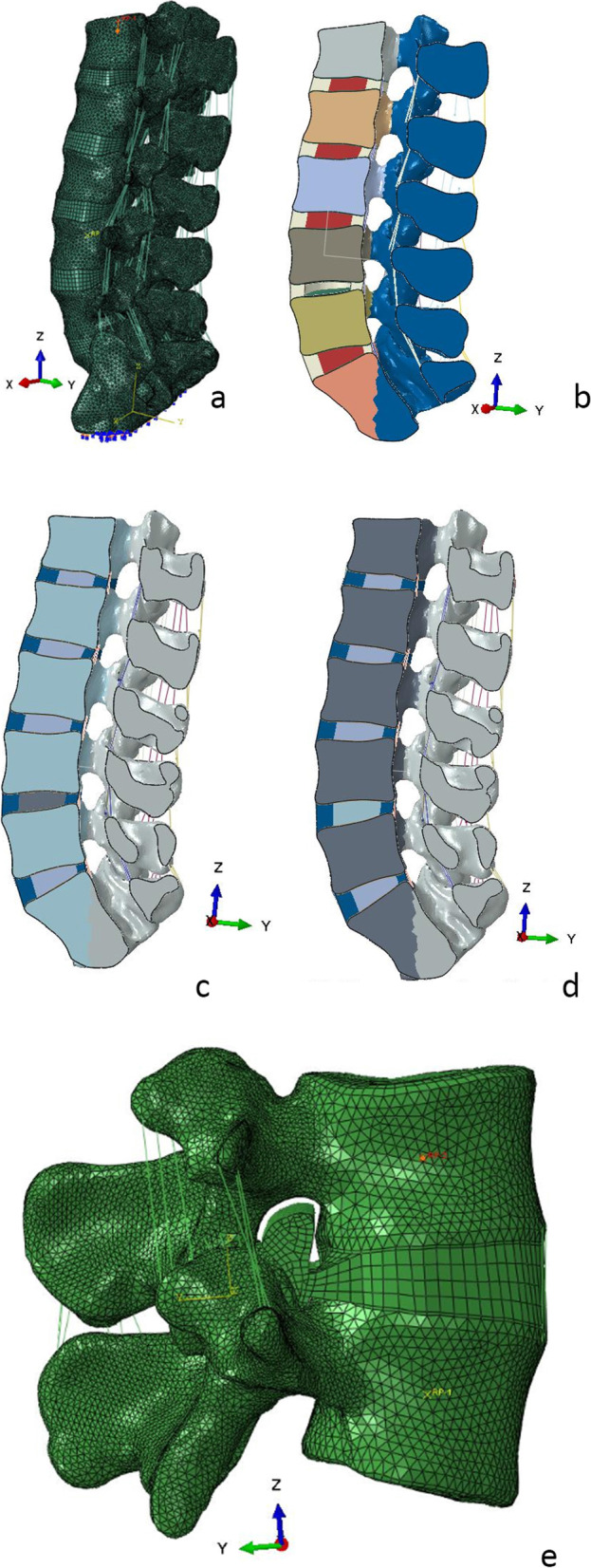
Finite element model. (**a**) N model (components include fibrous rings + nucleus pulposus). (**b**) H model: the intervertebral disc degeneration model (components include fibrous rings, cavitation the nucleus pulposus is removed in the simultaneous model). (**c**)H1 model:the intervertebral disc injected with 3 mL of bone cement model (components include fibrous rings + cement, cement is injected into the nucleus pulposus site). (**d**)H2 model:the intervertebral disc injected with 5 mL bone cement model (components include fibrous rings + cement, cement is injected into the nucleus pulposus site). (**e**) Nerve root model

Lumbar degeneration usually manifests as a decrease in disc height [19]. The model construction method described above was adopted to establish 3D digital models for patients with decreased disc height and cement injection. The main established models were as follows: normal lumbar standards (baseline model, N); intervertebral disc degeneration model (the L4-L5 disc degeneration with facet joints as the axis degeneration of approximate 5 degrees, the intervertebral foramen height was reduced, the disc nucleus pulposus cavitation had a degeneration height, H); the model H1 with the intervertebral disc injected with 3 mL of cement (for the site of nucleus pulposus cavitation in the L4-L5 disc, cement was injected, and the intervertebral height was increased degeneration height by ½ H, H1 = H + ½H, H1); the model H2 with the intervertebral disc injected with 5 mL of cement (for the site of nucleus pulposus cavitation in the L4-L5 disc, cement was injected, and the intervertebral height was increased by H, H2 = H + H, H2). The models are presented in Fig. 1. Disc components can be divided into four models: model N (components include fibrous rings + nucleus pulposus), degeneration model H(components include fibrous rings, cavitation the nucleus pulposus is removed in the simultaneous model), cement model H1, H2 (components include fibrous rings + cement, cement is injected into the nucleus pulposus site).

### Establishment of the finite element model

According to the calculated data and the numerical model of the lumbar spine, the actual biological structure was inferred. The corresponding parameters were evaluated for the lumbar vertebrae mesocortical bone, cancellous bone, cartilage final edition, posterior structure (vertebral arch, transverse process, and spinous process), fibrous ring, nucleus pulposus, and various ligaments (anterior and posterior longitudinal ligaments, supraspinous ligaments, interspinous ligaments, ligamentum flavum, joint capsule ligaments, and intertrochanteric ligaments) for relevant mechanical simulation. The ligaments were set as truss elements subjected only to tensile load. Based on the bioanatomic features, the ligament link locations in the model were connected to the ligament attachment points using the ANSA software, and a nonlinear truss unit (no compression) was built to simulate the ligament structure considering the biomechanical structural state of the ligament. The material properties of each tissue structure and implant equipment in the L1-S1 spinal segments analyzed in the present experimental study, along with the corresponding data from previous studies [20–23], are presented in Table 1. Considering the interactions of articular process joints in lumbar motion and the existence of lubricity between articular process joints, both upper and lower facet joint contact pairs were set for all facet joint sites and smooth frictionless were set in the simulation model.

**Table 1:** Material properties of the finite element model

Components	Young’s modulus (MPa)	Poisson ratio
Cortical bone	12,000	0.3
Cancellous bone	100	0.2
Vertebral arch	3500	0.25
transverse process	3500	0.25
spinous process	3500	0.25
Cartilage endplate	4000	0.3
Nucleus pulposus	1	0.49
Annulus	4.2	0.45
Anterior longitudinal ligament	20	0.3
Posterior longitudinal ligament	20	0.3
Ligamentum flavum	19.5	0.3
Interspinous ligament	11.6	0.3
Supraspinous ligament	15	0.3
Transverse ligament	58.7	0.3
Capsular ligament	32.9	0.3
Neurotissue sensor	20	0.3
Sacral vertebral	12,000	0.3
Bone cement	3000	0.3

The computational models were established using the ANSA software. The models mainly used tetrahedral units, hexahedral units, shell units (cortical bone), trusses units (ligaments), and a portion of the transition units, which were locally encrypted to ensure computational accuracy and computational speed. Discs (containing nucleus pulposus and annulus fibrosus), as well as endplates, with augmented hourglass controlled the three-dimensional hexahedral subtraction integral c3d8R (hourglass control reduces volume self-locking produced by large deformations of the unit). The hexahedral unit adopted the C3D8R cell type, the tetrahedral shell unit adopted the S4R cell type, the tetrahedral unit adopted the C3D4 unit, and the truss element adopted the T3D2 unit, accounting for a total of 504,757 solid units, 108,229 shell units, and 165 truss units for the entire model. A hexahedral mesh was employed because, in the same order, they have higher accuracy versus a triangular shell mesh versus a tetrahedral mesh with less computational expense. The reduced integral cell is adopted because this cell type can effectively reduce the generation of the cell “hourglass” phenomenon under the large deformation working condition, preventing the convergence of the calculation results due to the shear self-locking of the cell.

### Contacts

The upper and lower facet joint contact pairs were selected for all facet sites. The inter-articular lubrication was set to smooth and frictionless. In order to ensure the ease of loading, the coupling points were established at the center point of the L1 upper endplate, while the coupling of the associated upper face and sampling were placed at the L4 type center. The reference point for the coupling was L4. The forward flexion, backward extension, left turning, right turning, left flexion, right flexion, and right flexion movements of the lumbar spine were selected as the working conditions in the analysis. Loading was performed separately on different models for calculations. The present report describes the loading in every model through the application of upright 400 N and 10000n*mm loads on the coupled reference points while constraining the sacrum to all degrees of freedom. Load sharing was accounted for in our models, with a 400 N vertical and 10,000 n*mm torsional load applied in each active direction at the simulation model L1 position. The direction of lumbar activity is divided into forward flexion, backward extension, left turning, right turning, left flexion, and right flexion. The analysis of the working condition simulates real-life lumbar activity.

### Model validation

The validation of the model was compared with the findings of previous studies. Model N was subjected to 400 N concentrated force and 10000n*mm loads, and its ROM was measured under states of motion: flexion, extension, lateral bending, and axial rotation.

### Key observations

The relative mobility of the lumbar spine (range of motion, ROM) was determined based on the L4/5 segment angular displacement. The stress cloud plot derived the cartilage endplate, cement, nerve root stress, and the maximum stress value in each motion state. Using these indicators, the neural tissue-equivalent model was constructed and regarded as a stress sensor (the neural model was quite complex for this) to extract the pattern of the effects of the variation in the intervertebral foramen in different groups on the neural tissue.

## Results

### Model validation

The finite element model of the spine in the normal control model of the experiment had ROM of the L4/5 segment under different movement states, and the difference compared to the data in the literature was less (Table 2, 3)[20–25], which confirmed that the finite element model established in the present study had validity under certain conditions (Model was subjected to 400 N concentrated force and 10,000 n * mm loads).

**Table 2:** The comparison of L4/5 segment activity ranges among the normal spine model (Model N) and the study of Yamamoto et al.

Motion state	Model N	Yamamoto et al	Xiao et al
FE(°)	12.977	14.8 ± 2.10	14.20
LRB(°)	8.068	12.2 ± 2.25	13.23
LRR(°)	6.21	3.7 ± 1.50	4.23

*FE* Flexion and Extension, *LRB* Left and Right Bending, *LRR* Left and Right Rotation

**Table 3:** The comparison of L4/5 segment activity ranges among the normal spine model and the study of Shim et al.

Motion state	Model N	Shim et al
Extension(°)	5.45	2.79 ± 0.42
Flexion(°)	7.52	5.48 ± 0.88
Bending(°)	4.034	4.45 ± 1.01
Rotation(°)	3.105	3.80 ± 0.99

### Relative activity of the lumbar spine

The ROM values for the L4/5 segment of each model under different operating conditions are presented in Table 4. The ROM values of all the motion states of each finite element model are designated as H2, H1, H, and N, from small to large. Among these, the H2 ROM was the smallest among the three groups of surgical models and was the closest to the value for H1. The degrees of freedom in all directions decreased in the present situation of the degeneration state: the higher flexion and extension were approximately 95% of the normal benchmark, the higher flexion and extension were approximately 88% of the normal benchmark, and the right and left flexion was approximately 89% of the normal benchmark. The freedom of rotation in all directions after cement infusion into H1 was significantly reduced: the forward flexion extension was approximately 62% of the normal benchmark, the left–right rotation was approximately 47% of the normal benchmark, and the left–right flexion was approximately 50% of the normal benchmark. The freedom of rotation in all directions after cement injection into H2 was also significantly reduced: the forward flexion extension was approximately 63% of the normal benchmark, the left–right rotation was approximately 45% of the normal benchmark, and the right and left flexion was approximately 49% of the normal benchmark Normal model (baseline).

**Table 4:** The comparison of L4/5 segment activity ranges among the four spine models: N, H, H1, H2

L4-L5 Motion	Model	Simulation value (radian)	Relative ratio (combined)
**FE (x)**	N	0.22649	100% (Normal)
	H	0.21524	95.03%
	H1	0.14050	62.03%
	H2	0.14268	63.0%
**LRR (z)**	N	0.10839	100% (Normal)
	H	0.09513	87.8%
	H1	0.05082	46.9%
	H2	0.04919	45.4%
**LRB(y)**	N	0.14095	100% (Normal)
	H	0.12596	89.4%
	H1	0.07048	50.0%
	H2	0.06904	49%

*FE* Flexion and Extension, *LRB* Left and Right Bending, *LRR* Left and Right Rotation

### Stress peaks for cartilage endplate, nerve root, and cement for each finite element model

**Fig. 2:**
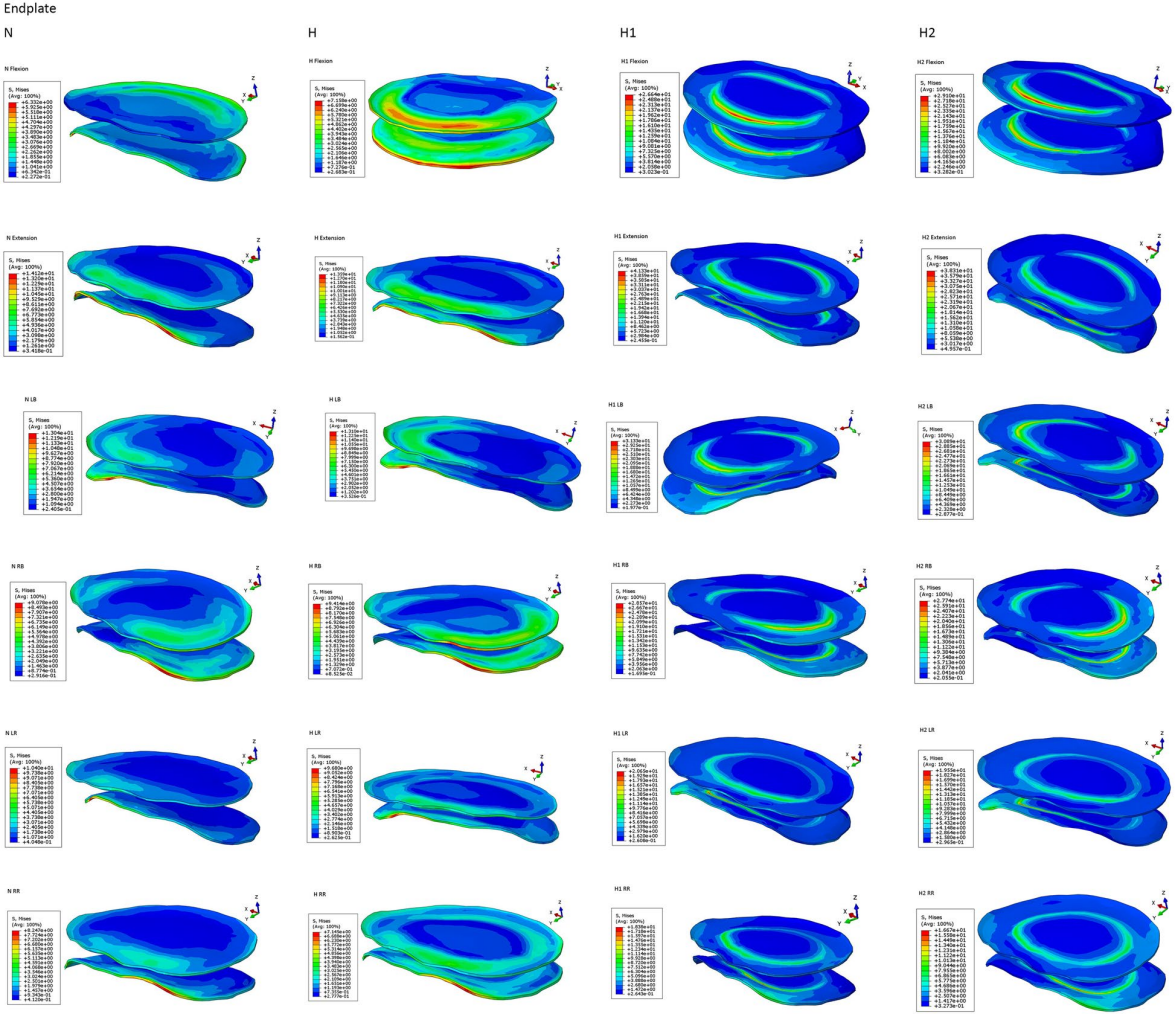
(**N**) Endplate stress distribution of N model in six motions. (**H**) endplate stress distribution of H model in six motions. (**H1**) endplate stress distribution of the H1 model in six motions. (**H2**) endplate stress distribution of H2 model in six motions. LB, Left Bending; RB, Right Bending; LR, Left Rotation; RR, Right Rotation

**Fig. 3:**
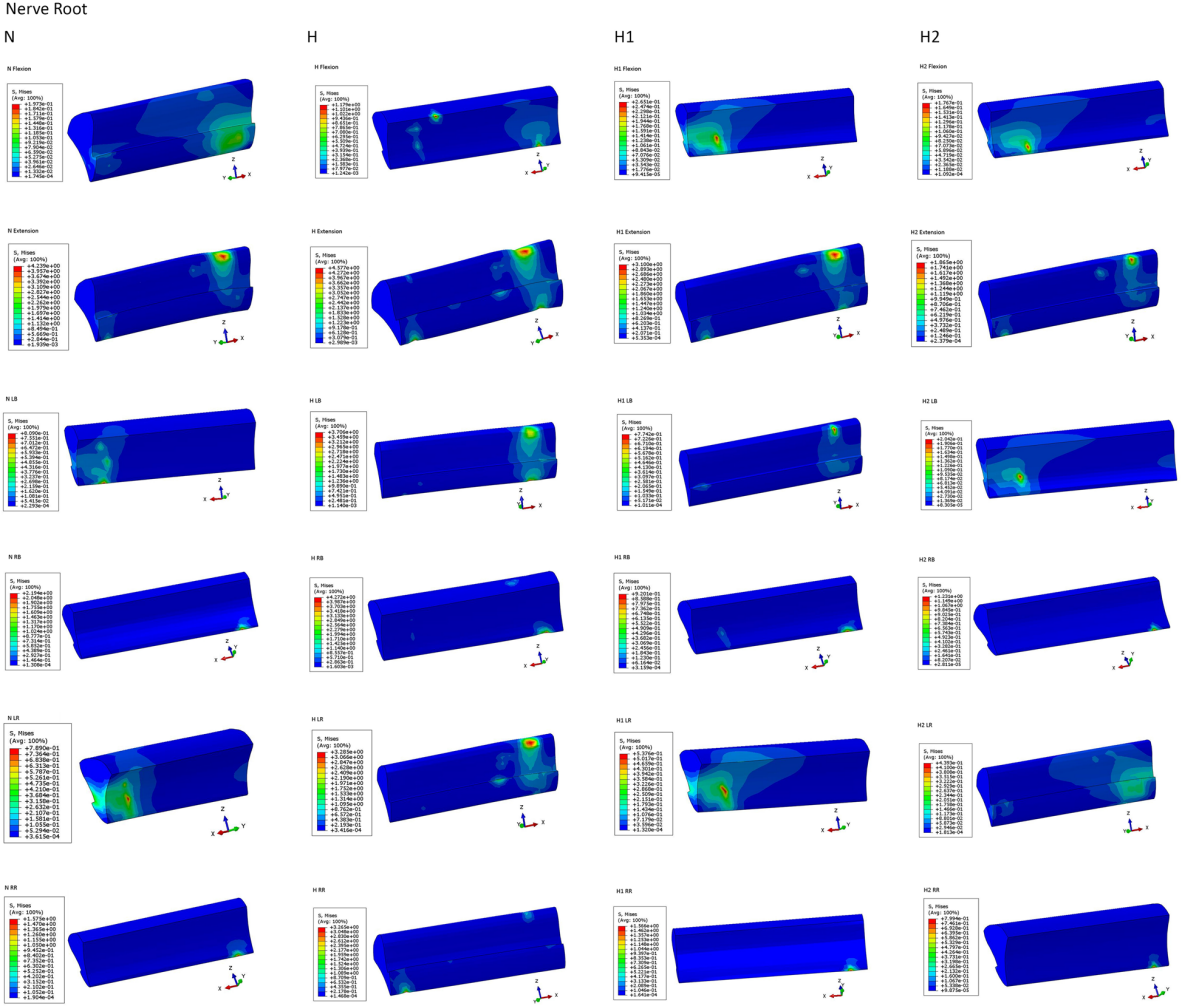
Nerve root stress distribution, (**N**) nerve root stress of N model distribution in six motions. (**H**) nerve root stress of H model distribution in six motions. (**H1**)the intervertebral disc injected with 3 mL of bone cement model nerve root stress of H1 model distribution in six motions. (**H2**) nerve root stress of H2 model distribution in six motions. LB, Left Bending; RB, Right Bending; LR, Left Rotation; RR, Right Rotation

**Fig. 4:**
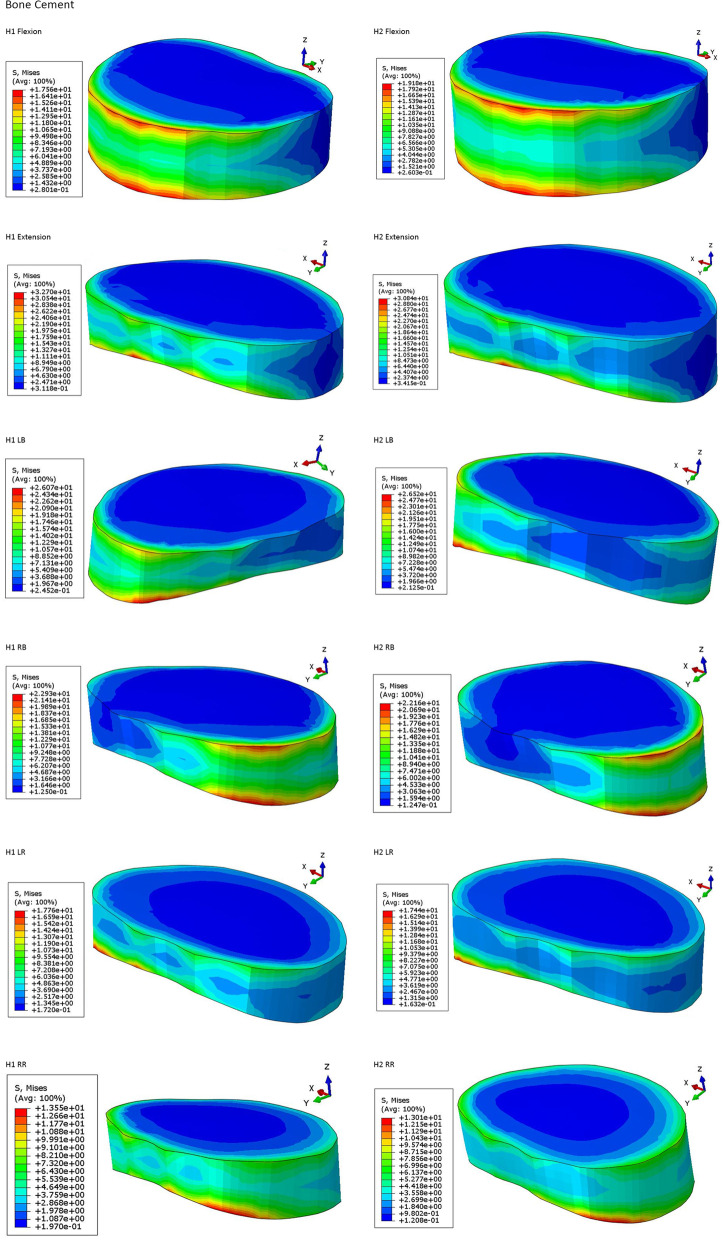
Bone cement stress distribution, (**H1**)bone cement stress distribution of H1 model for six motions. (**H2**)bone cement stress distribution of H2 model in six motions. LB, Left Bending; RB, Right Bending; LR, Left Rotation; RR, Right Rotation

The stress peaks for cartilage endplate, nerve root, and cement are presented in Table 5,6,7 and Fig. 2,3,4,5. The stress peaks for the cartilage endplate in the normal model (baseline, N) changed little after degeneration, while after injection of cement, the cartilage endplate stress increased significantly. In the two sets of cemented data, most data indicated a slight decrease in the endplate stress with increased amounts of injected cement. The stress for the cartilage endplate in the H1 model increased significantly during extension, left–right rotation, and left–right bending at 293%, 299%, 202%, 240%, and 315% of the benchmark, respectively. The stress for the nerve root in the H model was the largest. The stress for the nerve root in the H2 model was the smallest during flexion, extension, left–right bending, left rotation, right rotation at 90%, 44%, 25%, 56%, 56%, and 51% of the normal benchmark, respectively. After degeneration, each finite element model revealed decreased intervertebral height and compression of the surrounding tissue and nerve tissue upon rotation in all directions compared to the compression trend in the normal model. After the injection of bone cement, the nerve root stress is reduced. The greater the amount of cement, the higher the intervertebral height, and the lesser the nerve root stress. The peak value of cement stress in both the models changed little, exhibiting no evident trend.

**Table 5:** Stress peaks of cartilage endplate (Mise, MPa), four models: N, H, H1, and H2

	**N/Baseline**		**H/R**		**H1/R**		**H2/R**	
Flexion	6.332	100%	7.158	113%	26.64	420%	29.10	460%
Extension	14.12	100%	13.59	96%	41.33	293%	38.31	271%
LB	13.04	100%	13.10	100%	31.33	240%	30.89	237%
RB	9.078	100%	9.414	104%	28.57	315%	27.74	306%
LR	10.40	100%	9.680	93%	20.65	199%	19.55	188%
RR	9.088	100%	7.29	80%	18.38	202%	16.67	183%

*LB* Left Bending, *RB* Right Bending, *LR* Left Rotation, *RR* Right Rotation, *R* Relative ratio

**Table 6:** Stress peaks of nerve root (Mise, MPa), four models:N,H,H1,H2

	**N/Baseline**		**H/R**		**H1/R**		**H2/R**	
Flexion	0.1973	100%	1.179	598%	0.2651	134%	0.1767	90%
Extension	4.239	100%	4.577	108%	3.100	73%	1.8650	44%
LB	0.8090	100%	3.706	458%	0.7742	96%	0.2042	25%
RB	2.194	100%	4.272	195%	0.9201	42%	1.231	56%
LR	0.7890	100%	3.285	416%	0.5376	68%	0.4393	56%
RR	1.575	100%	3.265	207%	1.566	99%	0.7994	51%

*LB* Left Bending, *RB* Right Bending, *LR* Left Rotation, *RR* Right Rotation, *R* Relative ratio

**Table 7:** Stress peaks of bone cement (Mise, MPa), two models: H1 and H2

	**H1/R**		**H2/R**	
Flexion	17.56	100%	19.18	109%
Extension	32.70	100%	30.84	94%
LB	26.07	100%	26.52	102%
RB	22.93	100%	22.16	97%
LR	17.76	100%	17.44	98%
RR	13.55	100%	13.01	96%

*LB* Left Bending, *RB* Right Bending, *LR* Left Rotation, *RR* Right Rotation, *R* Relative ratio

**Fig. 5:**
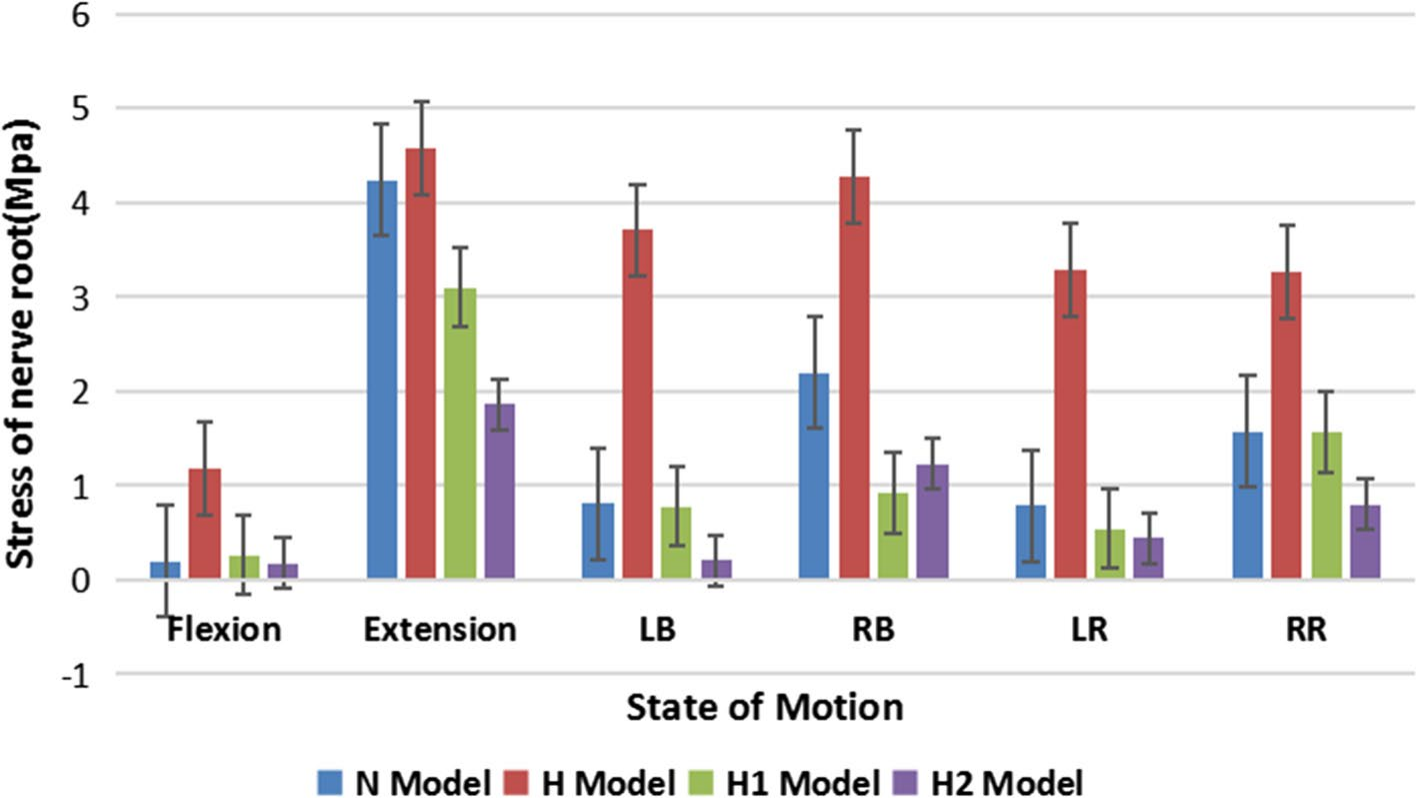
Nerve root stress distribution, (**N**) nerve root stress of N model distribution in six motions. (**H**) nerve root stress of H model distribution in six motions. (**H1**) nerve root stress of H1 model distribution in six motions. (**H2**) nerve root stress of H2 model distribution in six motions. LB, Left Bending; RB, Right Bending; LR, Left Rotation; RR, Right Rotation

## Discussion

PCD, with the primary treatment objective of reducing highly degenerative discogenic pain, is indicated for patients unable to undergo open surgery and those having imaging evidence for the disc vacuum phenomenon. Degenerative spine pathology occurs due to aging or biological changes in the intervertebral disc leading to morphological abnormalities. Degenerative spine pathology leads to changes in the imaging observations, with most cases exhibiting a decrease in the disc height, the collapse of the disc appearing in MR imaging, the disappearance of the nucleus pulposus structure in the CT of the spine, and other visible signs of disc vacuum [26, 27]. As the disc space height decreases, the size of the neural foramen decreases continuously or dynamically. Biomechanically, the degree of foraminal stenosis worsens in the standing or sitting position, while the size of the foramen increases in the lying position [28]. Periodically repeated compression of nerve roots may lead to chronic radiculopathy and cause localized and radiating pain in the setting of axial loading. Clinically, the patient complained of severe low back and leg pain which worsened when the patient was in the upright position and walking and reduced when the patient was in a recumbent position. The percutaneous discoplasty design principle is based on segmental instability, damage to the neural foramen area while standing, reduction in its effective area, and decompression in a recumbent position. Application of bone cement to fill a vacuum disc and the percutaneous injection of PMMA through the disc cavity provide prompt segmental stabilization effect and indirect decompression to alleviate clinical symptoms. The main objectives of minimally invasive PCD surgery are to reduce pain and restore patients' quality of life. PCD has demonstrated excellent clinical outcomes in patients unable to receive spinal fusion and those who have severe medical diseases. Therefore, PCD may be considered a minimally invasive and reasonable treatment option. PCD would provide segmental stabilization, foraminal decompression, and lumbar deformity correction as well [29–31].

The number of biomechanical studies conducted for PCD remains low to date. This study investigated the indirect decompression effect of cement with different doses on nerve roots and the biomechanical changes on the spine during PCD using finite element analysis (FEA). The result shows that the stress of the nerve root in model H was the largest. The nerve root stress in the model H2 was the smallest during flexion, extension, left bending, right bending, left rotation, right rotation at 90%, 44%, 25%, 56%, 56%, and 51% of the normal benchmark, respectively. After the injection of bone cement, the nerve root stress is reduced. The nerve root stress was smaller in Model H2 than in Model H1. The greater the amount of cement, the lesser the nerve root stress. We analyzed the stress changes of the degenerative intervertebral disc during bone cement filling into disc space and also analyzed the stress of bone cement between H1 and H2. The peak value of cement stress in both the models changed little, exhibiting no evident trend. The results of the study showed that the intervertebral height recovered after cement injection and the effect of indirect decompression of nerve root was obvious because the intervertebral height has been defined in the model as decreasing after degeneration and recovering after cement injection, but also after different doses of cement were injected into the intervertebral disc position, the change of stress on the surface of cement in the two groups did not much illustrate that cement is invisible. It can maintain good deformation and maintenance of intervertebral height. These results were consistent with the findings reported by Techens et al. They performed flexion extension, extension, and lateral flexion tests on 10 porcine lumbar segments, and each specimen in the experiment was evaluated under three conditions: intact disc, after nucleus pulpotomy, and after discoplasty. Studies have demonstrated that discoplasty restored posterior disc height and could open neural foramina, although without any effect on the mobility or stiffness of the spine [14]. In a study, the biomechanical changes occurring in cadaveric specimens after nucleus pulpectomy were compared with those occurring after PCD treatment, and it was concluded that discoplasty restored 80% of the disc height due to simulated flexion degeneration and 36% of the disc extension, thereby assisting in restoring the foramen width [32]. The motion was reduced in the present case of models H, H1, and H2, and the differences between model H1 and model H2 existed. Cartilage endplate stress was less in model H2 than in model H1. In the present study, the ROM values in the motion states of each Finite element (FE) model were designated as H2, H1, H, and N, from the smallest to the largest. The results could be attributed to the filling of intervertebral space with cement, which changed the elastic modulus of bone cement-filled disc tissue. These results were consistent with the findings reported by Wilke et al. [33].

While the finite element analysis approach offers several advantages over in vitro experiments when studying spine biomechanics, the model constructed using this approach is not perfectly aligned with the human structure. For instance, the individual variability of the model itself, no consideration of the muscle during modeling, and the structural trade-off between force analysis and morphology warrant further examination. Human tissues comprise complex bioactive structures, the material properties of which had to be studied by referring to the parameters reported in the foreign literature. At the same time, the actual numerical comparison may also exist. The intervertebral disc is a typical nonlinear structure with varying morphological changes. When it is modeled using a linear structure, the determined mechanical properties could be different from those in the actual scenario within the human body. The present model used constructs rather than facet articular cartilage, while wire spring constructs replaced ligaments. Whether this kind of human structural substitute, which discards the original morphological basis and has good mechano-transduction, requires verification using different models. Since the results calculated using the finite element model reflected a certain transient state of patients in their postoperative period. In contrast, the process through which the human spine structure changes is complicated by the interference of several factors such as natural degeneration. The calculated results might only reflect the trends and not the actual changes. Therefore, relevant data from the model-based study must be integrated with the results of in vitro experiments to produce inferences that would serve as an accurate reference for clinical research and treatment. This is the main inadequacy of theoretical research. More factors should be considered, such as the amount of vacuum and the presence of clinical symptoms, with mechanical low back pain being the most important during PCD. The best candidates for discoplasty are cases with vacuum phenomenon and subchondral sclerosis, 2B, and especially 3B types [34]. More limitations of the study include lack of reliability since patients have different grades of degeneration and vacuum phenomenon.

## Conclusions

The nerve root stress increased after degeneration but decreased after intervertebral height recovery through cement injection, which resulted in a significant indirect decompression effect. The stress of the nerve root decreased with the increase in the amount of cement injection. The present study results would serve as a reference for the future clinical implementation of PCD.

## Data Availability

The datasets used and analyzed during the current study are available from the corresponding author on reasonable request.

## Abbreviations

PCD: Percutaneous Cement Discoplasty
FEA: Finite Element Analysis
PMMA: Polymethylmethacrylate
CT: Computed Tomography
3D: 3 Dimensions
N: Normal model N
H: Model H
H1: Model H1
H2: Model H2
LB: Left Bending
RB: Right Bending
LR: Left Rotation
RR: Right Rotation
FE: Flexion and extension
LRR: Left and right rotation
LRB: Left and right Bending
R: Relative ratio
